# White matter microstructure pathology in classic galactosemia revealed by neurite orientation dispersion and density imaging

**DOI:** 10.1007/s10545-014-9780-x

**Published:** 2014-10-25

**Authors:** Inge Timmers, Hui Zhang, Matteo Bastiani, Bernadette M. Jansma, Alard Roebroeck, M. Estela Rubio-Gozalbo

**Affiliations:** 1 Department of Cognitive Neuroscience, Maastricht University, PO Box 616, 6200 MD Maastricht, The Netherlands; 2 Department of Pediatrics, Maastricht University Medical Center, PO Box 5800, 6202 AZ Maastricht, The Netherlands; 3 Department of Computer Science and Centre for Medical Image Computing, University College London, Gower Street, WC1E 6BT London, UK; 4 Maastricht Brain Imaging Center (M-BIC), PO Box 616, 6200 MD Maastricht, The Netherlands; 5 Laboratory Genetic Metabolic Diseases, Maastricht University Medical Center, PO Box 5800, 6202 AZ Maastricht, The Netherlands

## Abstract

White matter abnormalities have been observed in patients with classic galactosemia, an inborn error of galactose metabolism. However, magnetic resonance imaging (MRI) data collected in the past were generally qualitative in nature. Our objective was to investigate white matter microstructure pathology and examine correlations with outcome and behaviour in this disease, by using multi-shell diffusion weighted imaging. In addition to standard diffusion tensor imaging (DTI), *neurite orientation dispersion and density imaging* (NODDI) was used to estimate density and orientation dispersion of neurites in a group of eight patients (aged 16–21 years) and eight healthy controls (aged 15–20 years). Extensive white matter abnormalities were found: neurite density index (NDI) was lower in the patient group in bilateral anterior areas, and orientation dispersion index (ODI) was increased mainly in the left hemisphere. These specific regional profiles are in agreement with the cognitive profile observed in galactosemia, showing higher order cognitive impairments, and language and motor impairments, respectively. Less favourable white matter properties correlated positively with age and age at onset of diet, and negatively with behavioural outcome (e.g. visual working memory). To conclude, this study provides evidence of white matter pathology regarding density and dispersion of neurites in these patients. The results are discussed in light of suggested pathophysiological mechanisms.

## Introduction

White matter (WM) pathology has been repeatedly observed in classic galactosemia (OMIM 230400), an inherited disorder of galactose metabolism due to severe galactose-1-phosphate uridylyltransferase (GALT, EC 2.7.7.12) deficiency (Holton et al 2001). Neonates develop acute symptoms following galactose ingestion. A galactose-restricted diet resolves the acute symptoms, but long term complications still occur in these patients, including language production impairments, speech (motor) abnormalities, slower information processing, memory and executive functioning deficits, and generally a lower intelligence level (Waggoner et al 1990; Antshel et al 2004; Potter et al 2008; Doyle et al 2010; Potter 2011; Timmers et al 2012; Waisbren et al 2012; Rubio-Agusti et al 2013).

The first extensive study on magnetic resonance imaging (MRI) appearance revealed signal hyperintensities on T2-weighted images in the majority of patients’ peripheral cerebral and cerebellar WM (Nelson et al 1992). Further, mildly enlarged lateral ventricles (in 33 % of the patients) and signs of cerebellar atrophy (in 13 %) were observed. The authors suggested that the abnormal signal intensity might be due to a primary abnormality in the biochemical structure of myelin secondary to deficient galactocerebrosides. Histopathological and biochemical examination in an untreated adult patient revealed low galactocerebroside levels, supporting this idea (Haberland et al 1971). Later studies continued to observe WM abnormalities (Crome 1962; Wang et al 2001; Otaduy et al 2006; Hughes et al 2009), and links with affected myelination (Böhles et al 1986; Widhalm et al 2002).

To quantitatively investigate WM pathology, diffusion-weighted imaging (DWI) can be used for assessing properties and potential abnormalities in WM tissue microstructure. By modelling the diffusion of water molecules, many different parameters can be estimated. Most widely known is fractional anisotropy (FA), based on diffusion tensor imaging (DTI). FA concerns the degree of anisotropic diffusion, which is higher in WM (because of coherently formed fibre bundles) compared to grey matter (GM). In numerous diseases, reductions in FA have been found and linked to axonal degeneration (e.g. in amyotrophic lateral sclerosis, Chapman et al 2013) or myelin breakdown (e.g. in multiple sclerosis, Roosendaal et al 2009). FA has been shown to be very sensitive, but is inherently non-specific (Pierpaoli et al 1996), as a reduction could be caused by a reduction in neurite (axons and dendrites) density, an increase in dispersion of orientation, and several other factors. To disentangle two key contributors to FA, an approach called *neurite orientation dispersion and density imaging* (NODDI) was developed (Zhang et al 2012). By distinguishing three compartments in the brain (intra-, extra-neurite and cerebral spinal fluid) that are each modelled in a biologically informed manner, the neurite density and orientation dispersion can be estimated and analysed individually. In WM and GM, these indices have shown great correspondence to histological measures such as optical myelin staining intensity (Jespersen et al 2010), and quantitative Golgi analysis (Jespersen et al 2012), respectively. In WM, orientation dispersion quantifies bending and fanning of axons, and changes in neurite morphology have been implicated in diseases, although still mainly studied histologically in post-mortem tissue. In multiple sclerosis, for instance, axonal loss — reflected by reductions in axonal density and area — has been found in normal appearing WM (Evanglou et al 2000). The correlation between FA and neurite density, however, is relatively weak, suggesting that for diseases primarily affecting axonal density neurite density might be a more sensitive marker of pathology compared to FA. Successful and reliable estimation of neurite density and orientation dispersion has been shown in previous studies (Assaf and Basser 2005; Zhang et al 2012), and a recent clinical study has demonstrated its usefulness in localisation of cortical malformations in epilepsy patients (Winston et al 2013). In the current study, we applied NODDI to a patient cohort with classic galactosemia to study WM microstructure and establish relationships with the observed cognitive profile.

## Methods

### Participants

Eight patients with galactosemia and eight age- and gender-matched healthy controls participated in this study. Classic galactosemia was diagnosed by GALT enzyme activity assay and/or GALT-gene mutational analysis, and all patients adhered to a galactose restricted diet. Characteristics of the groups, including behavioural measures from a previous study (visual and verbal working memory, sustained attention and voice onset times during a language production task; Timmers et al 2012), can be found in Table 1. Participants had no other relevant health conditions, were screened for MRI compatibility and signed informed consent (in the case of minors, both parents/caregivers also gave written informed consent). The Medical Ethical Committee of the Maastricht University Hospital/Maastricht University gave ethical clearance for this study.

**Table 1:** Participant characteristics

	Patients		Controls	
	value	range	value	range
group size	8		8	
males / females	2 / 6		2 / 6	
age (in years) ^1^	17.9	15.9–21.2	17.2	14.7 – 20.0
GALT activity (in % of reference value) ^2^	0.54 %	0–1.52 %		
GALT mutation	4	Q188R/Q188R		
	2	400Tdel/unknown		
	1	L195P/K229N		
urine galactose concentration (in μmol/mmol creatinine)	11.9	ND ^3^-33		
urine galactitol concentration (in μmol/mmol creatinine)	140	97-187		
age at initiation of diet (in days)	11.8	0–35		
visual working memory (t-score) ^4^	32.3	22–51		
sustained attention (mean RT in seconds) ^5^	13.8	11.3–18.1		
verbal working memory (scaled score) ^6^	3.9	3–7		
voice onset time sentence production (in seconds) ^7^	1.97	1.49–2.20		

^1^ Age did not differ significantly between the groups [*F*_1, 16_ = 0.44, *p* = 0.519]. ^2^ GALT activity was measured at diagnosis; ^3^ ND = not detectable; ^4^ as assessed by Rey Osterrieth Complex Figure Immediate recall (Meyers and Meyers 1995); ^5^ as assessed by mean reaction time in Bourdon-Vos task (Vos 1988); ^6^ as assessed by Digit Span subtest of WISC-R [scaled score has mean of 10, SD of 3] (van Haasen et al 1986); ^7^ average voice onset time in a sentence production task (see Timmers et al 2012, for more information on these behavioural measures)

### Data acquisition

Data were acquired on a 3-T Siemens Trio whole body scanner (Siemens Healthcare, Erlangen, Germany), using a 32-channel head coil. The DWI data were obtained using a doubly refocused single-shot spin echo EPI sequence. Sixty four slices [voxel-size 2.2 mm isotropic; repetition time [TR] = 8500 ms; echo time [TE] = 97 ms) were acquired at two different b-values: b = 1000 and 2000 s/mm^2^, both with 64 diffusion-encoding gradient directions. In addition, five b = 0 images were collected, two of which were acquired using a reversed phase encoding direction (posterior to anterior). Total acquisition time for the sequence was 22.5 minutes.

### Data analyses

Pre-processing of the data included estimating susceptibility induced distortions. From the pairs of images with reversed phase-encode directions (and thus distortions going in opposite directions), the susceptibility-induced off-resonance field was estimated (similar to Andersson et al 2003; topup of FMRIB Software Library [FSL] version 5.0, Smith et al 2004). Further, eddy current-induced distortions and subject motion were estimated, and all distortions were corrected (using FSL's *eddy*). B-vectors were rotated to account for the corrections (using Python; http://www.python.org).

From one shell of the corrected DWI data (b = 1000 s/mm^2^), diffusion tensors were estimated using a linear fitting algorithm (*dtifit*, FSL). Diffusion Tensor Imaging ToolKit (DTI-TK) was used for tensor-based spatial normalization of the volumes to an iteratively optimized population-specific template (Zhang et al 2006) (http://www.nitrc.org/projects/dtitk). The algorithm applies a deformable registration to the tensor images, resulting in improved registration compared to standard FA-based registration algorithms (Wang et al 2011; Keihaninejad et al 2013). The resulting normalized maps were averaged and high-resolution FA maps (1 mm iso-voxel) were derived. The mean FA map was thinned to create a mean FA skeleton, representing the centres of all WM tracts common to all participants (tract based spatial statistics [TBSS], FSL, Smith et al 2006). After skeleton evaluation, each participant's aligned data was then projected onto this skeleton using calculated distance maps, and the resulting data were fed into the statistical analysis.

In parallel, NODDI was applied to the pre-processed data (http://nitrc.org/projects/noddi_toolbox). The NODDI tissue model distinguishes between three compartments: 1) intra-neurite space, representing the neurites and modelled as restricted diffusion (*sticks*, incorporating orientation dispersion); 2) extra-neurite space, surrounding the neurites, modelled as hindered, but not restricted diffusion (anisotropic Gaussian diffusion); and 3) cerebral spinal fluid (CSF), modelled as isotropic Gaussian diffusion. The main resulting parameters are: neurite density index (NDI), derived from the intra-neurite volume fraction (high in WM, low in GM); and orientation dispersion index (ODI), quantifying variation of neurite orientation (ranging from 0 for perfectly coherently oriented structures, to 1 for isotropic structures; this index is typically high in GM, low in WM). The output scalar maps from NODDI were normalized to the —already defined— study-specific common group space using the transformation fields as calculated per participant during the tensor-based registration. Then, the normalized NDI and ODI data were projected onto the —already calculated— mean FA skeleton using the original distance maps.

On the skeletonised FA, NDI and ODI maps, permutation-based statistics were carried out (as implemented by *randomise* in FSL; 5000 permutations). First, a design was used having group as a between-subjects factor and age as a covariate. Second, correlations were calculated with age, several disease variables (i.e. age at onset of the diet, GALT enzyme activity) and available behavioural outcomes (visual and verbal working memory, sustained attention, voice onset times in a language production task) were examined across the skeleton (age only) and within regions of interest (ROIs). In the skeleton approach, *p*-values were corrected by means of the threshold-free cluster enhancement (TFCE) option (Smith and Nichols 2009). In the ROI approach, no correction for multiple testing was applied (due to the small sample size), but the effect size of the correlations was taken into account (only large correlations [*r* >0.5] were considered as relevant; Cohen 1988). An alpha of 0.05 (corrected for multiple comparisons where applicable) was used as the significance level.

## Results

### Differences across groups

**Fig. 1:**
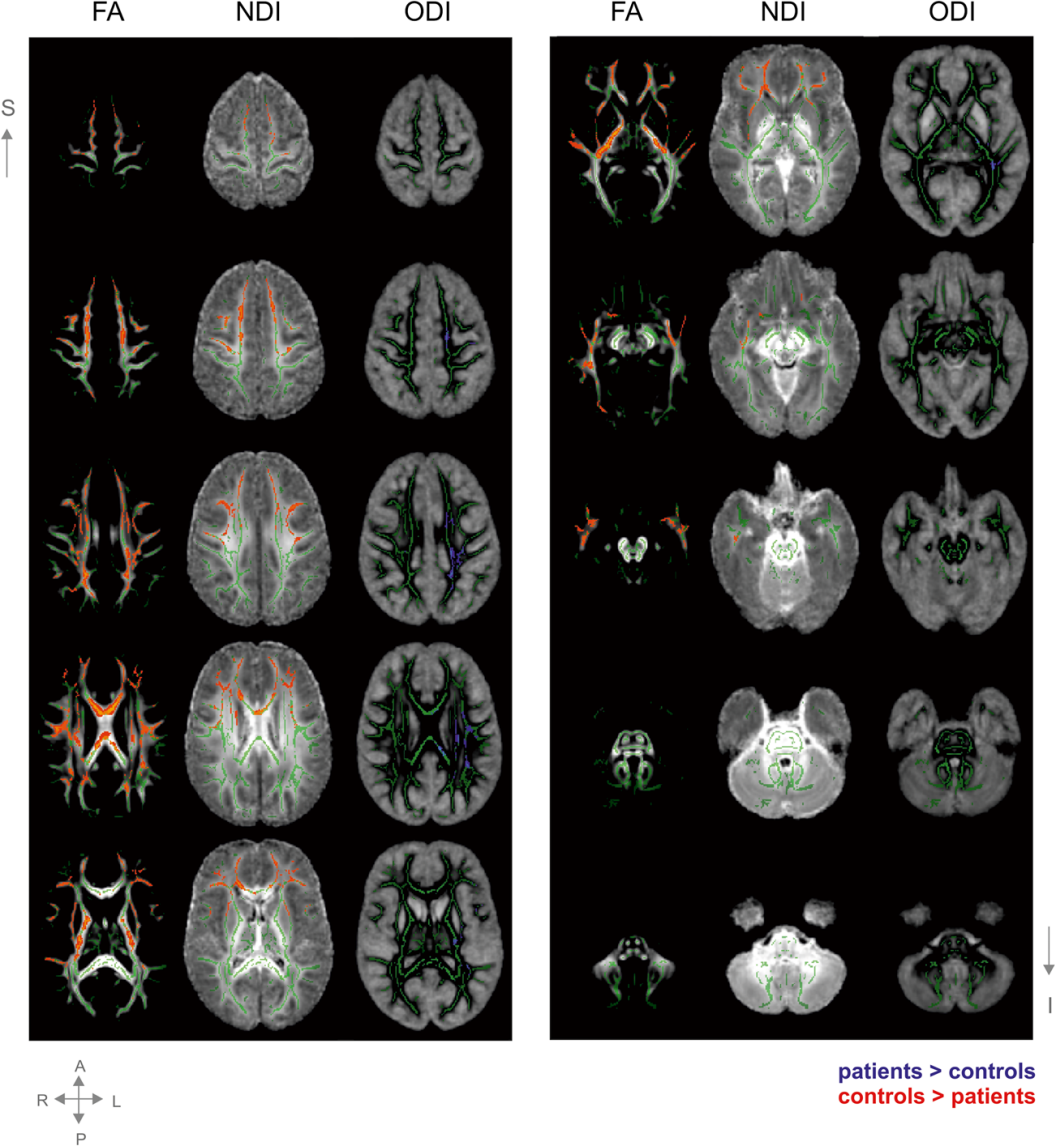
Group differences in main white matter tracts. Average group maps of the FA values, neurite density index (NDI) values, and orientation dispersion index (ODI) values (all ranging from 0.2-0.8) in transversal slices covering the majority of the brain from superior to inferior. Superimposed are the mean FA skeleton (green) and the statistical group differences (red: controls > patients; blue: patients > controls). Presented results are TFCE-corrected and thresholded at a corrected alpha-level of 0.05. For display purposes, the results are thickened by filling it out into the local tracts (as implemented in TBSS). Note that left is right in these maps

On the left columns of Fig. 1, mean FA maps are shown. Superimposed are the significant group differences, observed across the majority of the WM tracts, except for the cerebellar tracts.

**Fig. 2:**
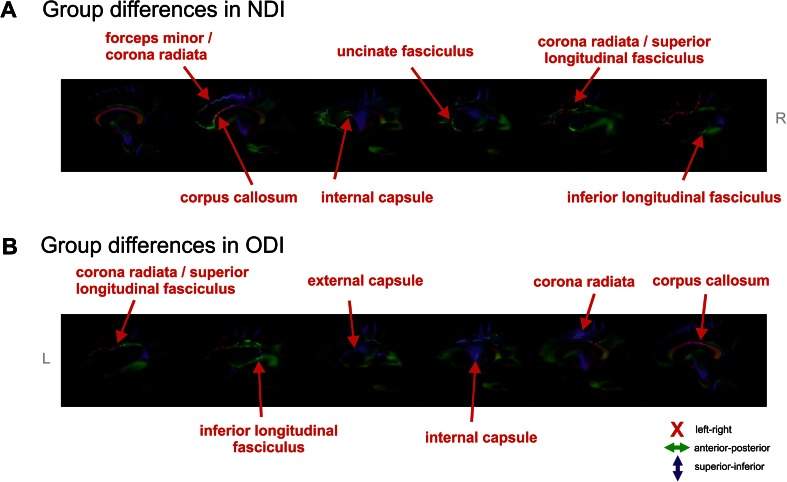
Sagittal colour coded maps of the mean diffusion tensor image. Red: left-right; blue: superior-inferior; green: anterior-posterior. Superimposed in white are statistical group differences from the NDI and ODI data. The red arrows identify the affected tracts. Affected tracts with respect to NDI include the anterior part of the corpus callosum (CC) and forceps minor (bilateral), corona radiata (bilateral), part of the internal (IC; right) and external capsula (EC; right), uncinate fasciculus (UF; bilateral), superior longitudinal fasciculus (SLF; bilateral) and inferior longitudinal fasciculus (ILF; right). Affected tracts with respect to ODI include the CC, corona radiata (bilateral) and IC (cortico-spinal tract), EC, SLF and ILF

In the middle columns of Fig. 1, the group differences in NDI are shown. Visual comparison shows these changes overlap with FA changes, but are more localized. NDI changes were found mainly bilateral and located mostly in the anterior part of the brain. In Fig. 2, the group differences are overlaid on colour maps, aiding in the localization of the tracts (using the atlas of Wakana et al 2003).

On the right-sided columns of Fig. 1, group differences in ODI are displayed, overlaid on mean ODI maps and the skeleton. Again, ODI changes overlap FA, but are more specifically localized. Dispersion changes were mainly located on the left, middle parts of the brain (see Fig. 2 for tract localisation) and showed minimal overlap with the NDI changes.

To examine the cerebellum irrespective of the skeleton, ROIs were manually drawn based on the group averaged FA map: two in bilateral middle cerebellar peduncles (one more anterior, one more posterior). No group differences were found in FA, NDI or ODI (all *p* > 0.3).

### Correlations with age, disease and behavioural variables

**Fig. 3:**
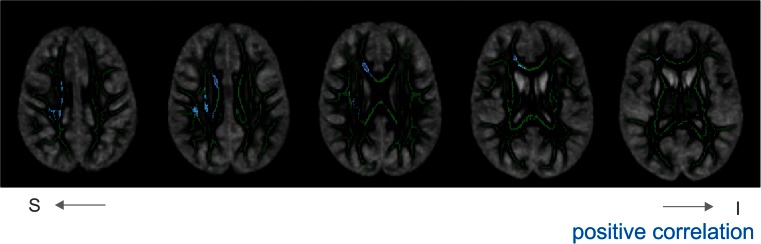
Correlations between ODI and age across the skeleton. Significant correlations are overlaid on the mean skeleton and mean ODI map. Depicted in blue are positive correlations (there are no negative correlations). Presented results are TFCE-corrected and thresholded at a corrected alpha-level of 0.05. For display purposes, the results are thickened. Note that left is right in these maps

FA, NDI and ODI did not correlate with age across the skeleton in the controls. ODI values in the patients, however, tended to be higher in older patients in several regions, mostly on the right hemisphere (Fig. 3). No correlations with age or behavioural measures were found for NDI and FA in the patients.

Areas showing significant group differences in NDI and/or ODI were used as regions of interest (ROIs). Associations between these ROIs with age, age at onset of diet, and GALT enzyme activity were calculated (Table 2; only correlations of *p* < 0.1 and *r* > .5 are reported). ODI in left CC was positively associated with age in patients (*r* = 0.77), while in controls it was negatively associated with age (*r* = −0.68). Further, the age at onset of diet showed several negative correlations: in forceps minor (with NDI and FA) and in EC (with FA).

**Table 2:** Region of interest correlation analyses

Regions of interest based on group differences in neurite density index (NDI)												
	NDI						FA					
	mean values (SD)		correlations				mean values		correlations *r*			
	patients	controls	patients		controls		patients	controls	patients		controls	
Left												
corpus callosum	0.61 (0.03)	0.67 (0.03)					0.65 (0.02)	0.69 (0.04)	age	−0.76 *		
									GALT act	0.64 ^+^		
forceps minor	0.51 (0.03)	0.59 (0.03)	age diet	−0.79 *			0.44 (0.02)	0.50 (0.02)	age	−0.62 ^+^		
corona radiate	0.57 (0.02)	0.63 (0.03)					0.53 (0.03)	0.59 (0.02)				
external capsule	0.52 (0.03)	0.57 (0.03)					0.52 (0.02)	0.47 (0.04)				
superior longitudinal fasciculus	0.62 (0.03)	0.67 (0.03)					0.46 (0.03)	0.52 (0.01)				
Right												
corpus callosum	0.62 (0.04)	0.69 (0.03)					0.67 (0.04)	0.72 (0.02)	age	−0.70 ^+^		
forceps minor	0.51 (0.03)	0.58 (0.03)	age diet	−0.78 *	age	−0.643 ^+^	0.46 (0.02)	0.52 (0.02)	age diet	−0.72 *		
corona radiate	0.58 (0.01)	0.62 (0.03)	visual mem	0.68 ^+^			0.67 (0.04)	0.72 (0.02)				
			sust attent	0.64 ^+^								
internal capsule	0.62 (0.02)	0.67 (0.03)					0.65 (0.03)	0.65 (0.04)	age diet	−0.65 ^+^	age	0.679 ^+^
external capsule	0.50 (0.02)	0.55 (0.01)					0.41 (0.03)	0.46 (0.02)	age diet	−0.73 *		
inferior occipital fasciculus	0.51 (0.03)	0.6 (0.02)					0.51 (0.04)	0.6 (0.04)				
inferior longitudinal fasciculus	0.55 (0.03)	0.6 (0.04)					0.52 (0.03)	0.58 (0.04)	age diet	−0.63 ^+^		
									visual mem	0.71 *		
superior longitudinal fasciculus	0.63(0.04)	0.68 (0.03)					0.48 (0.05)	0.53 (0.02)	age diet	−0.65 ^+^		
uncinate fasciculus	0.49 (0.03)	0.55 (0.02)					0.46 (0.03)	0.51 (0.03)				
Regions of interest based on group differences in orientation dispersion index (ODI)												
	ODI						FA					
	mean values (SD)		correlations				mean values (SD)		correlations *r*			
	patients	controls	patients		controls		patients	controls	patients		controls	
Left												
corpus callosum	0.08 (0.01)	0.06 (0.01)	age	0.77 *	age	−0.68 ^+^	0.75 (0.04)	0.81 (0.02)				
corona radiate	0.22 (0.01)	0.19 (0.01)	age	0.69 ^+^			0.50 (0.03)	0.56 (0.02)				
internal capsule	0.15 (0.01)	0.13 (0.01)					0.66 (0.02)	0.70 (0.03)				
external capsule	0.25 (0.01)	0.21 (0.01)	age diet	0.64 ^+^			0.40 (0.03)	0.46 (0.02)	VOT	−0.64 ^+^		
inferior longitudinal fasciculus	0.13 (0.01)	0.10 (0.01)					0.62 (0.05)	0.65 (0.02)			age	−0.74 *
superior longitudinal fasciculus	0.19 (0.01)	0.16 (0.02)	VOT	−0.68 ^+^			0.51 (0.05)	0.56 (0.04)			age	−0.75 *
Right												
corona radiate	0.22 (0.03)	0.19 (0.03)	age	0.68 ^+^			0.40 (0.06)	0.50 (0.03)	age diet	−0.65 ^+^		

*p < 0.05; ^**+**^ *p* < 0.10; age diet = age at initiation galactose-restricted diet; GALT act = residual GALT enzyme activity in % of reference value; visual mem = t-score at Rey Osterrieth complex figure immediate recall task (visual memory); sust att = mean reaction time at Bourdon-Vos task (sustained attention); VOT = voice onset time during language production task (see also Table 1)

**Fig. 4:**
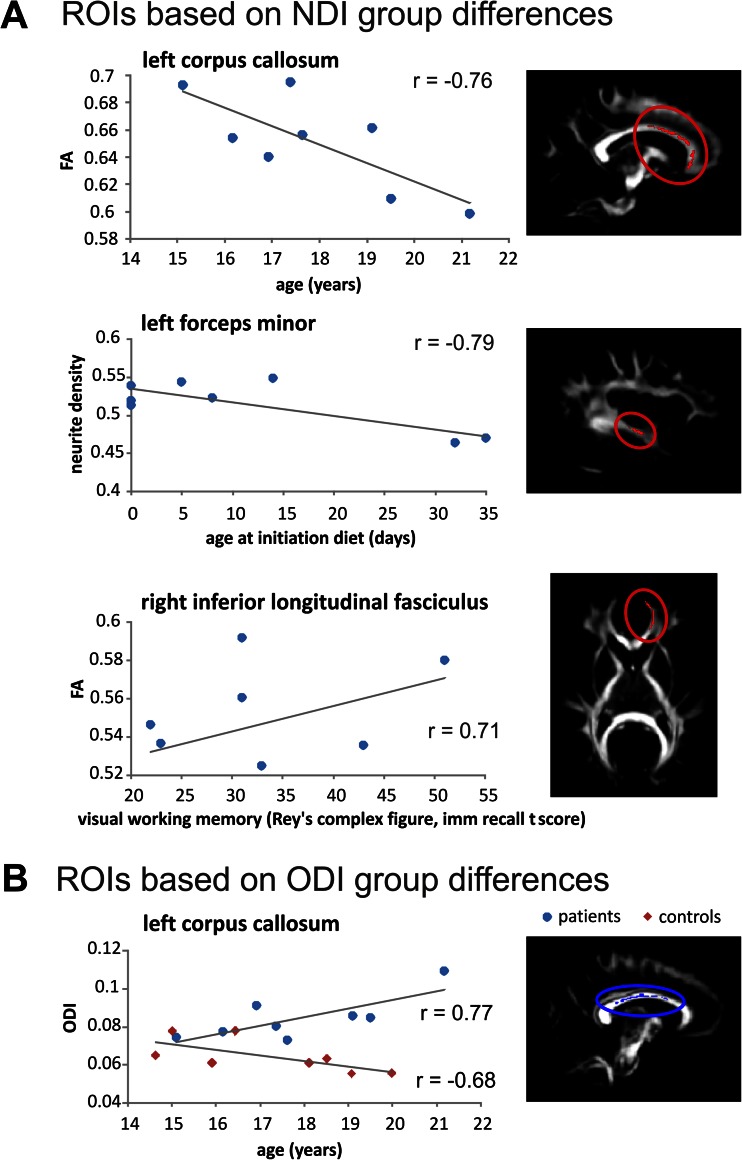
ROI correlation analyses. A selection of significant correlations of regions of interests (ROIs) based on group differences in NDI (**a**) or in ODI (**b**). Added are a linear trend line, the correlation and an illustration of the location of the ROI

Mean values in the ROIs were correlated with behavioural outcome measures of the patient group as well (i.e. visual and verbal working memory, sustained attention, voice onset times in a language production task). Better visual working memory performance was correlated with higher FA values in right ILF (*r* =0.71) (see Fig. 4 for a selection of correlations).

## Discussion

In the current study, we used NODDI to investigate WM properties of patients with classic galactosemia. Patients showed lower FA values, lower density (NDI) and higher orientation dispersion (ODI) of neurites in several tracts as compared to the control group. These indices showed significant correlations with age, disease variables and several behavioural outcome measures.

### WM pathology in the patients with classic galactosemia

Extensive differences between patients and matched controls were found. Standard DTI analyses showed lower FA values in the patient group spread over almost the entire cerebrum. The NODDI analysis allowed us to disentangle two main factors contributing to FA — NDI and ODI — and these measures showed more localized and specific results. Lower NDI was revealed mainly in the anterior parts of the brain (bilateral), and higher ODI was mostly left-lateralized.

Other studies have observed widespread WM abnormalities in these patients, with a tendency to be clustered around the lateral ventricles (Nelson et al 1992). We also observed clustering of abnormalities along the lateral ventricles, as SLF, ILF and IC run alongside this ventricle. In contrast to Nelson et al (1992), we observed involvement of both the CC and IC, suggesting that the current method is more sensitive to reveal abnormalities. We did not observe any abnormalities in the cerebellar WM, which is surprising as cerebellar abnormalities (atrophy and WM abnormalities) and impairments suggesting cerebellar damage have been observed in this disease (e.g. Nelson et al 1992; Potter et al 2013). However, we cannot exclude that there may be fine-grained differences that will be picked up with tract-specific analyses.

Lower neurite density in patients with classic galactosemia could be an indirect result of abnormal myelination. Although DWI cannot directly assess myelin, a reduction in myelin will result in lower NDI. In turn, this reduced density could lead to less coherently organized axons, and thus to increased ODI. Hence, increases in ODI could indirectly result from abnormal myelination as well. In WM, which in general is highly coherently oriented (as opposed to GM), higher dispersion may be less favourable. Besides abnormal myelination, increased ODI in these patients might be a result of reduced or delayed pruning of axons. In several regions, however, we observed that ODI increased with age, which does not fit with abnormal pruning (but rather with axonal sprouting). Noteworthy is that the correlations between ODI and age were mainly observed in the right hemisphere, while the group differences were largest in the left hemisphere. This apparent dissociation is, however, most likely a result of the limited statistical power due to the small sample size. Another explanation for the increased orientation dispersion is that it reflects increased branching or sprouting of axons as a compensation mechanism. Data from future longitudinal designs could give more insight in this matter.

### Observed abnormalities in relation to the cognitive profile

In general, the left-lateralized nature of the ODI findings is in accordance with the observed language and motor abnormalities in this disease, as both are generally left-lateralized (Kell et al 2011; Gotts et al 2013). We observed abnormalities in both the UF (NDI) and the SLF (NDI and ODI). Both tracts — or more specifically the UF and part of the SFL (also referred to as the arcuate fasciculus, AF) — are strongly associated with language processing (Glasser and Rilling 2008; Saur et al 2008; Friederici 2009). The AF connects temporal and parietal regions with the frontal lobe. Abnormalities in the AF have been linked to conduction aphasia (Catani and Mesulam 2008), and underdevelopment of this tract in children goes hand in hand with language processing difficulties (Friederici 2009). The finding that longer voice onset times in a language production paradigm (Timmers et al 2012) tended to be associated with higher orientation dispersion in the SLF is supportive of the relation with language production impairments. The UF provides a ventral route between inferior frontal regions and more anterior superior temporal regions, and has been associated with language functions as well (Saur et al 2008). In addition, we observed involvement of the IC that contains fibres projecting from the medulla oblongata to the cerebral cortex, and include motor tracts such as the corticospinal tracts. Further, abnormalities were found surrounding the precentral gyrus, the premotor and primary motor area of the brain. Together, these findings are in line with motor and motor speech abnormalities often reported in patients with galactosemia (Potter 2011; Shriberg et al 2011; Potter et al 2013; Rubio-Agusti et al 2013).

Furthermore, the anterior nature of the NDI changes and the extensive involvement of the corona radiata fit with a profile showing impairments in higher order processes. For instance, working memory functions are subserved by regions in the prefrontal and inferior frontal cortex, among other regions (Martin and Chao 2001; Cabeza et al 2002). Additionally, maturation of WM in several regions of the frontal lobe correlates with performance in working memory tasks (Klingberg 2006), and networks of attention have been shown to involve the SLF and anterior corona radiate (Ge et al 2013). In the current study, lower performance in behavioural tasks such as visual working memory and sustained attention tended to be associated with lower (less favourable) NDI and FA in the anterior corona radiata. Due to the explorative nature of the study and the limited available behavioural outcome measures, more extensive studies carrying out tract-specific analyses should be performed to confirm these relations.

### Relations of age and age at onset of diet with observed pathology

Several studies in galactosemia have suggested that the cognitive complications progress with age, while others have refuted this claim (Doyle et al 2010; Waisbren et al 2012). In the current study, we found several regions showing a positive correlation between age and ODI, meaning that ODI tends to be higher in older patients (but not in controls). Similar correlations with age were observed in the ROIs. Although we are mindful that our sample size was relatively small (adding to a risk for false positives, Button et al 2013), no correction for multiple testing was performed because of the small sample size (observed correlations were, nevertheless, large: *r* >0.7, interpreted using Cohen's convention, 1988), and the data are cross-sectional in nature, the results seem to indicate a progressive nature, at least with respect to the ODI changes. Furthermore, the present data suggests that the older the patient at the onset of the diet, the less favourable the WM properties in many tracts.

It remains unclear why certain regions are more affected than others. Compensation for impairments in language and motor cognitive domains could explain the localization of the ODI changes. NDI, on the other hand, is lower particularly in anterior regions. It is known that, during normal development, myelination starts in the posterior parts of the brain and spreads to anterior regions. In prefrontal regions myelination even continues into adolescence (Fuster 2008; Asato et al 2010). This late myelination could be more disturbed in this disease. Future studies to understand the timing and precise mechanisms of the damage are warranted.

## Conclusion

We demonstrated WM microstructure abnormalities in patients with classic galactosemia in both the density and the orientation dispersion of axons. Specific regional profiles were found that are in agreement with the cognitive profile in galactosemia: a left-lateralized profile in ODI in line with language and motor abnormalities, and an anterior pattern of NDI changes in accordance with the general profile of higher order cognitive impairments. In addition, correlations of the WM properties with age, age of onset of the diet and with behavioural outcome such as visual working memory were revealed. Further studies involving larger samples and more extensive neuropsychological assessments are needed to confirm the current findings and to explore the relations between affected tracts and cognitive (dys)functioning in more depth.

## Abbreviations

AF: Arcuate fasciculus
CC: Corpus callosum
CSF: Cerebral spinal fluid
DTI: Diffusion tensor imaging
DTI-TK: Diffusion tensor imaging ToolKit
DWI: Diffusion-weighted imaging
EC: External capsule
FA: Fractional anisotropy
FSL: FMRIB Software Library
GALT: Galactose-1-phosphate uridylyltransferase
GM: Grey matter
IC: Internal capsule
IFL: Inferior longitudinal fasciculus
MRI: Magnetic resonance imaging
NDI: Neurite density index
NODDI: Neurite orientation dispersion and density imaging
ODI: Orientation dispersion index
ROI: Region of interest
SLF: Superior longitudinal fasciculus
TE: Echo time
TFCE: Threshold-free cluster enhancement
TR: Repetition time
UF: Uncinate fasciculus
WM: White matter
